# Ear biometrics for patient identification in global health: a cross-sectional study to test the feasibility of a simplified algorithm

**DOI:** 10.1186/s13104-016-2287-9

**Published:** 2016-11-02

**Authors:** Elizabeth J. Ragan, Courtney Johnson, Jacqueline N. Milton, Christopher J. Gill

**Affiliations:** 1 Department of Global Health, Boston University School of Public Health, Boston, MA USA; 2 Center for Global Health and Development, Boston University School of Public Health, Boston, MA USA; 3 Department of Biostatistics, Boston University School of Public Health, Boston, MA USA

## Abstract

**Background:**

One of the greatest public health challenges in low- and middle-income countries (LMICs) is identifying people over time and space. Recent years have seen an explosion of interest in developing electronic approaches to addressing this problem, with mobile technology at the forefront of these efforts. We investigate the possibility of biometrics as a simple, cost-efficient, and portable solution. Common biometrics approaches include fingerprinting, iris scanning and facial recognition, but all are less than ideal due to complexity, infringement on privacy, cost, or portability. Ear biometrics, however, proved to be a unique and viable solution.

**Methods:**

We developed an identification algorithm then conducted a cross sectional study in which we photographed left and right ears from 25 consenting adults. We then conducted re-identification and statistical analyses to identify the accuracy and replicability of our approach.

**Results:**

Through principal component analysis, we found the curve of the ear helix to be the most reliable anatomical structure and the basis for re-identification. Although an individual ear allowed for high re-identification rate (88.3%), when both left and right ears were paired together, our rate of re-identification amidst the pool of potential matches was 100%.

**Conclusions:**

The results of this study have implications on future efforts towards building a biometrics solution for patient identification in LMICs. We provide a conceptual platform for further investigation into the development of an ear biometrics identification mobile application.

**Electronic supplementary material:**

The online version of this article (doi:10.1186/s13104-016-2287-9) contains supplementary material, which is available to authorized users.

## Background

One of the greatest public health challenges in low- and middle-income countries (LMICs) is identifying people over time and space, that is, identifying people at repeated time points regardless of when or where. The success of our major efforts, including chronic infectious disease management, vaccination campaigns, and longitudinal studies, hinges upon accurate identification at point of initial care and then correct re-identification from there on out.

Finding a simple and reliable system to identify and track individuals in LMICs over time and space is one of the most pressing public health challenges of our day. Recent years have seen an explosion of interest in developing electronic medical records (EMRs) and information technology (IT) systems for hospitals and health care centers in LMICs [1–6]. Yet electronic records offer no benefit over paper records if one cannot accurately identify a given individual. With widespread mobile phone ownership and access to network signal, mobile health technology, or mHealth, is uniquely poised to address this problem [7, 8].

It was with this challenge in mind that we began to investigate the possibilities for a simple, cost-efficient, and portable mHealth solution to subject identification. Biometrics is a method of recognizing individuals via unique physiological attributes [9], is an advancing field for person identification, and has promise for application in mHealth. Biometrics is becoming an increasingly popular means for identification on an international level. Large-scale international systems for purposes of immigration, verifying identity, controlling restricted access areas, and controlling restricted information are now active in the United Kingdom (UK), Belgium and other European Union countries [10]. India’s Aadhaar biometric ID programme is also worth noting, which has been established with the goal of enrolling all adults into the system to aid in the delivery of social welfare programs [11]. However, these systems are prime examples of the general trend of biometric application for person identification; they are large-scale, complex, multi-scalar, and generally inappropriate as analogs for our settings of interest.

We developed seven criteria that a chosen biometrics system would have to meet in order to solve the challenge of person identification in LMICs. First, it should operate on one of the more commonly used smart phone operating systems, such as Android or the Apple iOS system. Second, it must minimize data storage requirements on the device itself: in settings where cellular data transfer rates are slow and expensive, population data for subject identification will need to be stored locally on the cell phone itself. Third, it must be physically non-invasive and culturally acceptable. Fourth, it must be secure so that even if a phone was lost or stolen, subject confidentiality would not be compromised. Fifth, the system must be able to perform both identification and verification, recognizing returning patients and enrolling new patients as they enter into the system. Sixth, its design must be optimized for use in children. Lastly, it must be ‘sufficiently’ accurate to consistently recognize individuals through periods of rapid growth, such as the first year of a child’s life.

Most existing biometric targets fail to meet one or more of these criteria. We considered popular biometrics approaches, including finger and palm-printing, iris scanning, DNA testing, and facial recognition against our criteria, but deemed all to have at least one critical shortcoming. Iris scanning, for instance, requires a consistent light source (typically with infrared wavelengths), is comparatively expensive to other biometrics technologies, and requires that the subject keep their eye open for a specified duration of time—a particular challenge when dealing with small children who may be frightened by the scanner [12–14]. Fingerprinting, which currently takes up the largest proportion of biometrics application, is susceptible to finger pad damage (of particular concern among rural populations who partake in daily manual labor). Perhaps most importantly, fingerprinting recognition can carry a negative connotation with law enforcement, and this stigma alone renders fingerprints unacceptable to many of the individuals that are most in need [15, 16].

Ears met our criteria. First, the only technology required is a camera and a consistent image capturing process. Second, photos can be rendered into minimal data points allowing for local storage on a phone. Third, ears are easily accessible by non-invasive means and can be photographed without frightening an infant or young child. Fourth, ears are relatively impersonal features of our anatomy that tend not to mark the memory of others as distinguishing features, yet ears are sufficiently variable between individuals to serve as an identifier and are increasingly recognized as a viable biometric [16–27]. Lastly and most importantly, the ear is one of the most stable anatomical structures throughout the lifespan, already at approximately 75% of adult size at the time of birth, with linear, and therefore predictable, growth [18, 28].

For these reasons we hypothesized that ears would be an ideal biometric target for identification. To test this hypothesis, we first developed a simplified algorithmic system for extracting biometric measurements from photographs of individual’s ears. We explored various combinations of measurements, developing the algorithm based on the structures both supported by the literature and found to be consistently present yet sufficiently variable among our subjects. Second, we conducted a cross sectional study in which we photographed left and right ears from twenty-five adults, and then used our algorithm to extract the biometric measurements for each of the fifty ears. For analysis, we conducted a series of blinded re-identification experiments in which we used the derived algorithmic data to re-identify the subjects, using first data just from one ear, and subsequently data from paired left and right ears. This paper discusses the results of these investigations, providing proof of concept to justify the sophistication and digitalization of this simplified biometrics technique into a Smartphone application for patient identification.

## Methods

**Fig. 1:**
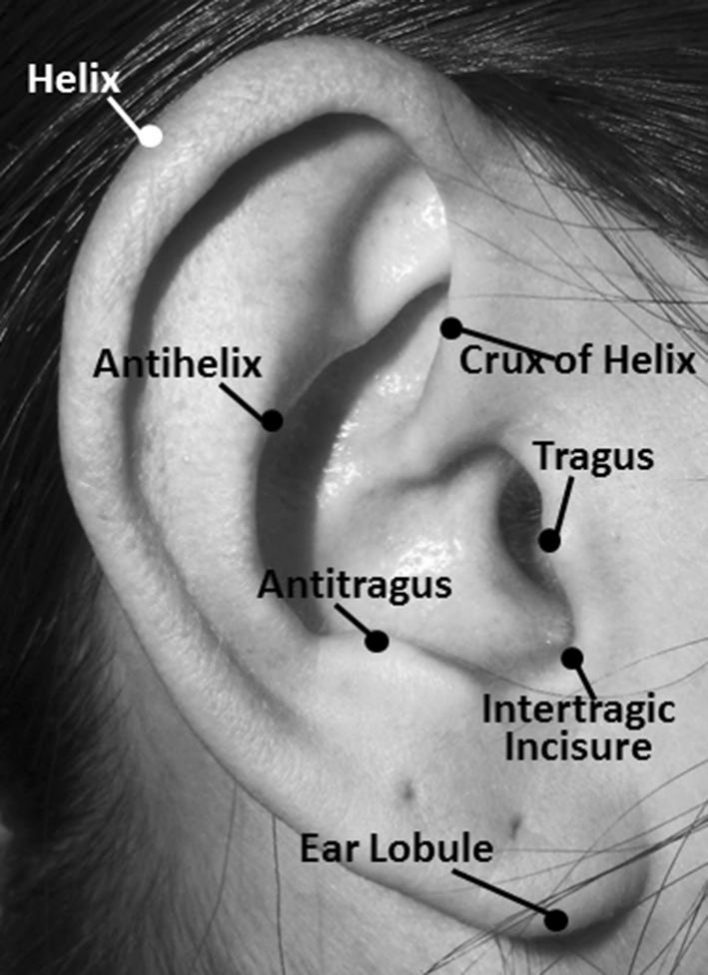
Anatomical ear structures used in algorithm development

To demonstrate proof of concept, our investigation evolved over two phases. First, using open-source photographs of ears, we evaluated a number of different approaches to converting image data into numerical formats algorithmically. The focus here was to both identify anatomical structures whose presence was relatively consistent between individuals and to identify structures that were convertible into numerical algorithmic representations. The anatomical structures we included in our investigation are highlighted in Fig. 1. The development of our algorithmic measurement method was partly derived from the literature on ear biometrics, but also guided empirically through trial and error of different combinations of features and measurements [24, 26, 29].

Second, we conducted a two-step validation study using images captured from human volunteers. The objective was to first test our ability to convert image data into numerical formats using the final algorithm from step one. We then used those numerical data to reconnect subjects to their identities. The algorithm validation study, approved by the Boston University Internal Review Board, was conducted in December 2013. We enrolled twenty-five adults from the Boston University Medical Campus who provided written consent (see Table 1). Photographs were taken of each individual’s left and right ears, yielding a total of fifty images. Ears were all photographed in the same interior lighting conditions with the flash enabled. We took care to ensure consistent alignment of the camera to the ear for the photograph, but without a stabilizing structure there was some variation in the angle at which the photograph was taken.

**Table 1:** Demographic descriptions of ear study participants

Variables	n = 25
Sex, %	
Male	9 (36%)
Female	16 (64%)
Age, years	
Mean (SD)	40.2 (13.0)
Min–max	23–68
Race, %	
White	17 (68%)
African American/Black	4 (16%)
Two or more	2 (8%)
Asian/Pacific Islander	1 (4%)
Other	1 (4%)

**Fig. 2:**
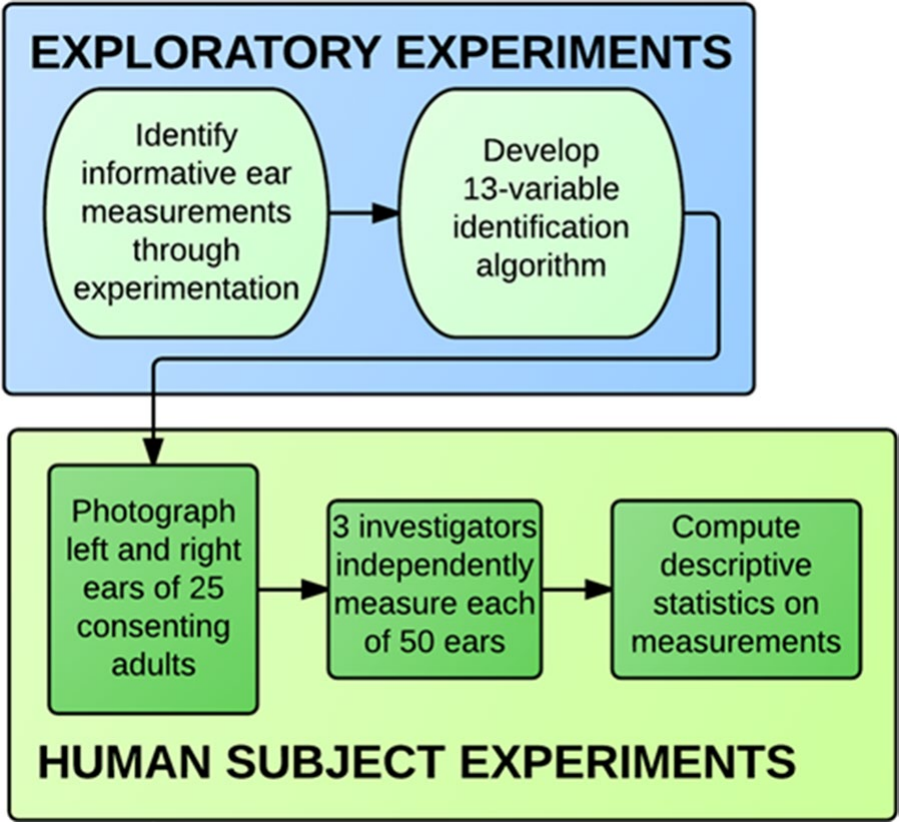
Process of algorithm development and data collection

For purposes of reducing error, left ear photographs were digitally reversed so that the tragus was on the right side of the photo and the outer edge of the ear was on the left side of the photo. Three investigators independently and systematically measured each ear to ascertain the base measurements for computing the identification algorithm. Lengths were measured in pixels with the program GIMP 2.8.10 and angles were measured with the program MB-Ruler 4.0 [30, 31]. As a result, the thirteen-component algorithm was captured independently for each of the left and right ears three times. The average for each algorithm measurement was computed. Standard deviations were calculated at the individual subject level, measuring the variation between each investigator’s value for each ear measurement, and also at the group level, measuring the variation for each algorithm variable across all subjects for each of the thirteen algorithmic variables. The ideal result would be that for any given algorithm variable for one specific ear, variation between investigators’ measurements would be minimal (i.e., standard deviation would be small), thus representing accuracy in the measurement process. Conversely, in order for this approach to be useful in correctly identifying an individual, we would hope to see considerable variation for a given algorithm variable between different subjects (i.e., larger standard deviations). The process of algorithm development and data collection can be seen in Fig. 2.

Once these calculations were made, we conducted two blinded re-identification experiments. For the first, left and right ears were treated as if from fifty different individuals. The purpose of this was first, to increase the sample size of our pool, and second, to test the sensitivity of the method while presuming that left and right ears, being genetically linked, would resemble one another. Each member of our team individually used a computerized random number generator to select forty of the fifty ears and redistribute those into random order. The associated forty average algorithms for each set of randomly selected forty ears were then blinded so that each team member was the only person who knew their true identity of the subjects in their set. We chose not to use all fifty ears to prevent the reviewer of the data from matching by process of elimination. We then swapped our lists and each attempted to use our own algorithm measurements to identify the correct match. That is, investigator A gave their randomly-ordered algorithms to investigator B. Investigator B gave their randomly-ordered algorithms to investigator C, and so on. Thus, we were testing not only the ability to distinguish one individual from the next, but also the ability to match an individual’s algorithm measurements back to the averages.

**Fig. 3:**
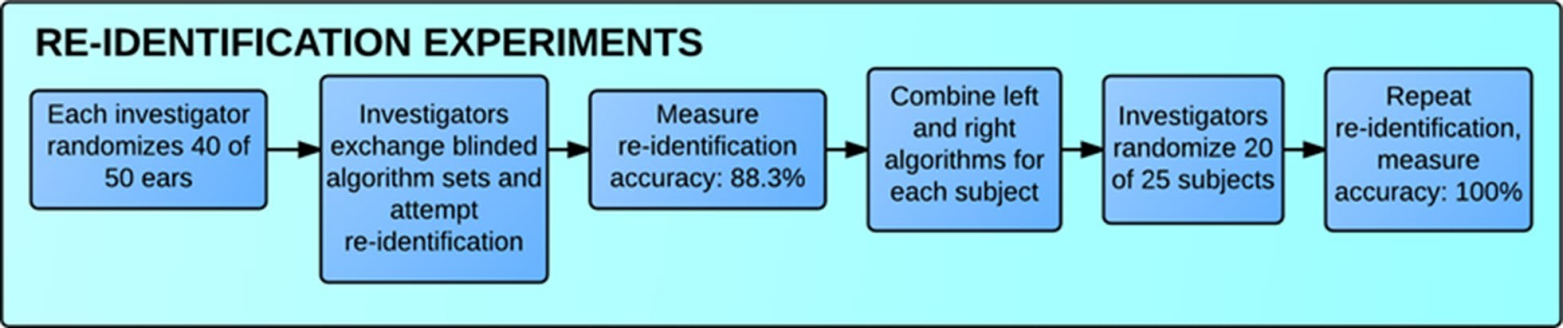
Process of two re-identification experiments, first considering left and right ears as independent entities, and second, combining the paired left and right ear algorithms for each subject to attempt to re-identify true identities

The second re-identification experiment kept the left and right ears of each individual together as a unique pair. This experiment paralleled true application, as in almost all circumstances both of an individual’s ears would be available to aid in identification and theoretically increase accuracy. Each investigator used the computerized random number generator to select twenty out of the twenty-five pairs (again to prevent matching by process of elimination) and redistribute them into random order, and the blinding experiment was repeated in the same fashion. The process of both re-identification experiments is depicted in Fig. 3.

Our primary statistical analysis was to conduct a principal component analysis (PCA) to determine the most meaningful variables of the algorithm, that is, the variables lending to the greatest inter-subject variability. Principal components are linear combinations of sets of variables from the given dataset at optimal weights, determined through the calculation of eigenvalues. Eigenvalues are used to consolidate variance in a correlation matrix, therefore the factor with the largest eigenvalue explains the most variance [32]. Therefore, because principal components are calculated based on eigenvalues, the first principal component accounts for the maximum amount of variance in the dataset, the second principal component accounts for remaining variance not accounted by the first principal component, and each successive principal component accounts for the diminishing remainder of variability still unaccounted for. A proportion of each variable entered into the analysis is described by each principal component. In the end, only the most meaningful principal components are retained [33]. PCA was conducted three times to parallel the logic in the re-identification analysis: first, on the thirteen variables of the left ears alone; second, on the thirteen variables of the right ears alone; and third, on the twenty-six combined left and right ear variables for each of the twenty-five subjects. We determined the number of principal components to retain through Kaiser’s eigenvalue-greater-than-one rule, which states that principal components are retained only if their eigenvalue is greater than 1.0, and Cattell’s scree plot method, which is a process of examining the eigenvalues of the principal components through a scree plot to identify where the line breaks and levels off (also called the ‘elbow’) which becomes the cut-off for inclusion [34, 35]. Additionally, we applied an orthogonal rotation to the retained principal components. We used SAS 9.3 software (SAS Institute, Cary NC) to conduct these analyses.

The study was approved by the ethical committee at Boston Medical Center and all subjects provided signed informed consent.

## Results

The process of identifying which anatomical structures to include in the algorithm highlighted the high variability between individuals. For example, while one individual may have a pronounced antitragus, another may have a convex structure instead. Additionally, one individual may have a distinct fold to the helix, while another may have an ear that apparently does not fold over at all. Even within a relatively small set of open-source ear images, it became apparent that very few structures could be depended upon from one person to the next and that the chosen algorithm would need multiple approaches in order to account for the likely absence of one or more structures.

The structures we based our final algorithm on included the helix, antihelix, tragus, antitragus, intertragic incisure, and the ear lobule (Fig. 1). The primary points of interest on each of these structures included what we termed the ‘anchor point’, or where the crux of the helix meets the antihelix perpendicularly, the most protruding point of the tragus and the antitragus, the maximum height from top of the ear to bottom of lobule, and the outer edge of the helix. The ‘anchor point’ proved pivotal in this analysis as it was the only anatomical feature that could be dependably identified on one subject from the next, and therefore became the starting point for the algorithm.

**Fig. 4:**
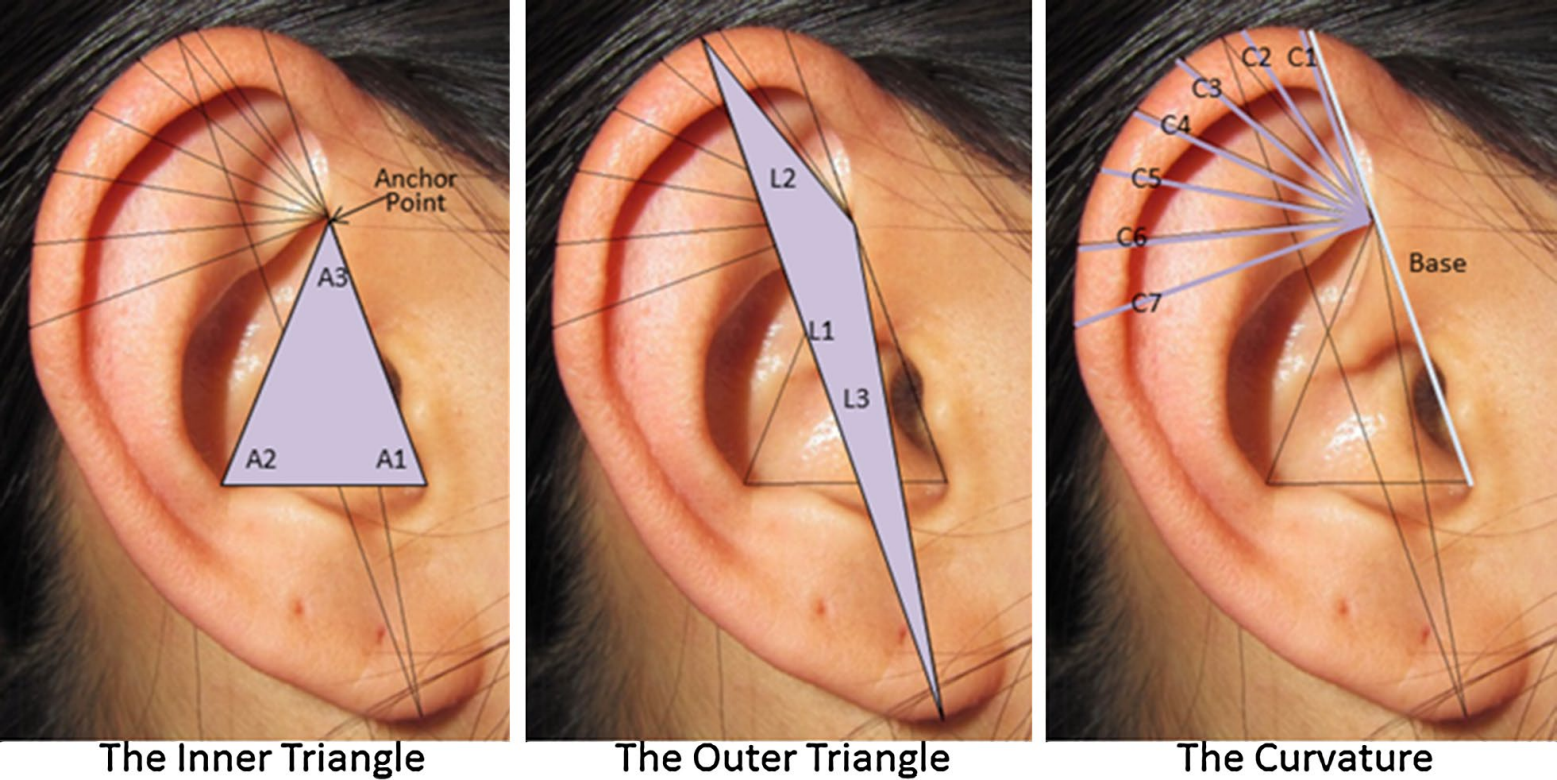
The three sections of the ear identification algorithm: The inner triangle, the outer triangle, and the curvature

The final algorithm was divided into three sections. The first, termed the ‘inner triangle’, consisted of a triangle beginning at the anchor point with the first edge running tangentially across the tragus until it met the base of the intertragic incisure, the second edge crossing tangentially over the antitragus until it reached the antihelix, and the third edge connecting back to the anchor point (Fig. 4). The second section, termed the ‘outer triangle’, was the maximum height of the ear, from the top of the helix to the base of the lobule (or in the case of attached earlobes, where the lobule ended in conjunction with the head), connected from both ends back to the anchor point. The last section was termed the ‘curvature’ and was made up of seven measurements taken at fifteen degree intervals rooted at the anchor point and measured to the outside of the helix, along with a base measure which ran from the intertragic incisure, through the anchor point, and up to the edge of the helix (or the end of C1) (Fig. 4).

In order to avoid the error introduced by variation in image resolution and/or distance between the ear and the camera at the point of image capture, we calculated ratios instead of absolute values. The first section of the algorithm was derived from the Inner Triangle, made up of three ratios between each of the three angles of the Inner Triangle. The second section was derived from the Outer Triangle, and was made up by the three ratios between each of the three triangle lengths. Although angles were preferred due to their robustness against changes in photo resolution, lengths were chosen for the outer triangle because the number of possible locations for the ends of the maximum height line led to more possible variance in the resulting ratios by angles than from absolute lengths. The final section of the algorithm was made up of seven ratio variables derived from seven curvature measurements at consecutive fifteen degree angles on the upper ear (C1 through C7), using the base line as the denominator in each case (see Fig. 4). For an outline of the algorithm ratio variables, see Table 2. Since our algorithm used ratios of two measurements (e.g. O1:O2), the absolute size of the image did not matter since proportionality was preserved. Rather, variability resulted from measurement errors and from the angle at which the image was taken. The former could be simply addressed by automating the process through programming. The latter problem could be reduced or negated by creating a mechanical cradle to hold the smartphone during image capture, effectively standardizing the distance from the camera to the subject’s head and the angle at which the image is taken.

**Table 2:** The ratio compositions of each algorithm variable, organized by the inner, outer, and curvature sections

Section	Variables	Ratio
Inner triangle	I1	A1:A2
	I2	A1:A3
	I3	A2:A3
Outer triangle	O1	L1:L2
	O2	L1:L3
	O3	L2:L3
Curvature	C_R_1	C1:base
	C_R_2	C2:base
	C_R_3	C3:base
	C_R_4	C4:base
	C_R_5	C5:base
	C_R_6	C6:base
	C_R_7	C7:base

To prove measurement accuracy between investigators, the standard deviations were calculated for each of the three investigator’s measurements for each algorithm variable and averaged across subjects. Accuracy would have been demonstrated by small standard deviations for each variable (Table 3). The mean standard deviation across all thirteen algorithm variables was 0.034 (left s.d.: 0.032; right s.d.: 0.036). However, it was observed that certain algorithm variables had markedly higher variation than others. The algorithm variables I2 and I3 of the inner triangle had high variation and I1 and O1 of the inner and outer triangles had moderately high variation. For this reason, we calculated standard deviations thrice more to analyze the sensitivity of the algorithm. First, we excluded what we determined to be the high variation variables (I2 and I3), and calculated the standard deviation to be 0.018 (left s.d.: 0.015; right s.d.: 0.022). Second, we additionally excluded the moderately high variation variables (I1 and O1) and calculated the standard deviation of the remaining nine variables to be 0.010 (left s.d.: 0.008; right s.d.: 0.013). Lastly, we calculated the standard deviation for only the seven curvature variables (C_R_1 through C_R_7), which was empirically observed to be the most accurate section of the algorithm, to be 0.009 (left s.d.: 0.007; right s.d.: 0.011). The variable with the overall highest accuracy was C_R_1, which was measured from the ‘anchor point’ to the top of the helix (Fig. 4), with a standard deviation of 0.007 (left s.d.: 0.006; right s.d.: 0.008). The variable with the least accuracy was I3 of the inner triangle (Fig. 4), with a standard deviation of 0.124 (left s.d.: 0.120; right s.d.: 0.129).

**Table 3:** Mean standard deviations (SD) of investigators’ measurements for each algorithm variable for both left and right ears to represent measurement accuracy, with analysis of sensitivity based on different variable combinations

Left ear algorithm			Right ear algorithm		
Variable	N	Mean SD	Variable	N	Mean SD
C_R_1	25	0.006	C_R_1	25	0.008
C_R_2	25	0.008	C_R_2	25	0.011
C_R_3	25	0.005	C_R_3	25	0.011
C_R_4	25	0.006	C_R_4	25	0.012
C_R_5	25	0.007	C_R_5	25	0.011
C_R_6	25	0.006	C_R_6	25	0.011
C_R_7	25	0.011	C_R_7	25	0.011
I1	25	0.035	I1	25	0.030
I2	25	0.127	I2	25	0.095
I3	25	0.120	I3	25	0.129
O1	25	0.061	O1	25	0.093
O2	25	0.007	O2	25	0.013
O3	25	0.012	O3	25	0.026
	Mean	0.032		Mean	0.036
		Overall mean SD = 0.034			
*Excluding I2 and I3*	Mean	0.015		Mean	0.022
		Overall mean SD = 0.034			
*Excluding I1, I2, I3, and O1*	Mean	0.008		Mean	0.013
		Overall mean SD = 0.010			
*Curvature only (C*_*R*_ *1*–*C*_*R*_ *7)*	Mean	0.007		Mean	0.011
		Overall mean SD = 0.009			

To test the effectiveness of differentiating one unique individual from the next, standard deviations for each average algorithm variable across subjects (that is, the average between the three investigators’ measurements) were calculated (see Table 4). The same algorithm variables that were deemed to have high inter-observer variation were found to have high inter-subject variation. We concluded this was a result of the low accuracy in measurement, and therefore we followed the same process of excluding variables in a sensitivity analysis. Across all twenty-five subjects, the average standard deviation for the thirteen algorithm variables was 0.212 (left: 0.247; right: 0.177). Excluding I2 and I3, the average standard deviation for the eleven variables was 0.096 (left s.d.: 0.102; right s.d.: 0.091). Additionally excluding I1 and O1, the average standard deviation for the nine variables was 0.062 (left s.d.: 0.062; right s.d.: 0.062). Finally, the standard deviation across the average algorithm measures for the seven curvature variables (C_R_1 through C_R_7) was 0.059 (left s.d.: 0.058; right s.d.: 0.059). The algorithm variables with the greatest variation across subjects reflected the variables with the least accuracy in measurement, with the highest being I3. Similarly, the algorithm variable with the least variation was C_R_1, which was the variable with the highest accuracy of measurement across investigators. However, the standard deviation across subjects was still over five-fold greater than the standard deviation between investigators, demonstrating its capacity to contribute to identification.

**Table 4:** Mean standard deviations (SD) of each algorithm variable across subjects for both left and right ears as a representation of ear variability across subjects, with analysis of sensitivity based on different variable combinations

Left ear algorithm			Right ear algorithm		
Variable	N	Mean SD	Variable	N	Mean SD
C_R_1	25	0.038	C_R_1	25	0.039
C_R_2	25	0.050	C_R_2	25	0.044
C_R_3	25	0.064	C_R_3	25	0.053
C_R_4	25	0.066	C_R_4	25	0.067
C_R_5	25	0.071	C_R_5	25	0.075
C_R_6	25	0.063	C_R_6	25	0.073
C_R_7	25	0.054	C_R_7	25	0.064
I1	25	0.204	I1	25	0.191
I2	25	0.886	I2	25	0.418
I3	25	1.205	I3	25	0.888
O1	25	0.353	O1	25	0.248
O2	25	0.073	O2	25	0.069
O3	25	0.080	O3	25	0.073
	Mean	0.247		Mean	0.177
		Overall mean SD = 0.212			
*Excluding I2 and I3*	Mean	0.102		Mean	0.091
		Overall mean SD = 0.096			
*Excluding I1, I2, I3, and O1*	Mean	0.062		Mean	0.062
		Overall mean SD = 0.062			
*Curvature only (C*_*R*_ *1*–*C*_*R*_ *7)*	Mean	0.058		Mean	0.059
		Overall mean SD = 0.059			

Re-identification experiments were conducted to test the function of the algorithm as whole to aid in distinguishing one individual from the next. For the first experiment, left and right ears were treated independently and each of the three investigators randomly selected forty out of the fifty possible subjects. From this set of 120 potential matches, we were able to precisely identify 88.3% of the blinded data sets. It is worth noting that almost all mismatches were the left or right counterpart of the correct ear and that the correct ear was always listed as a possible correct match by each investigator. If considering the expanded range\ of possibilities, 100% of the subjects were identified within a ranked list of the top three most likely candidates. The second re-identification experiment, where left and right ears were paired together, and each investigator randomly selected twenty out of the twenty-five possible subjects, we were able to precisely identify 100% of the subjects in the blinded sets. This was representative of the most likely scenario, as both left and right ears would almost always be available for analysis.

**Fig. 5:**
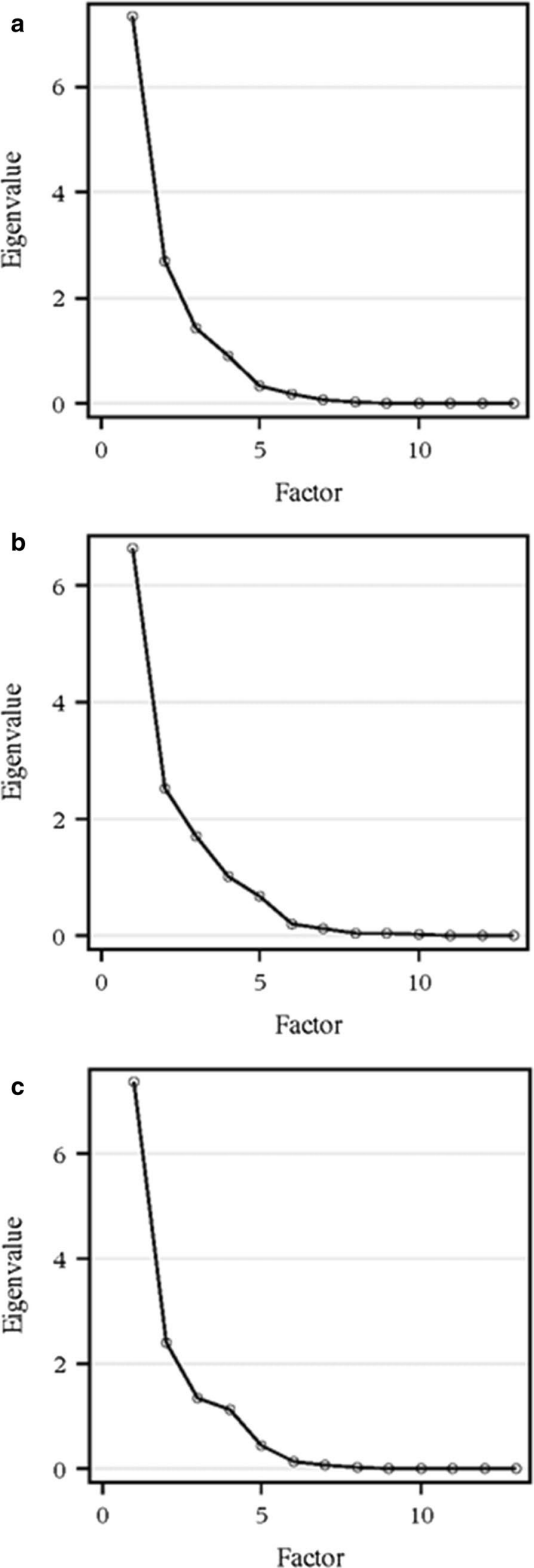
**a** Cattell’s scree *plot* for determining number of principal components for left ear PCA. **b** Cattell’s scree *plot* for determining number of principal components for right ear algorithm PCA. **c** Cattell’s scree *plot* for determining number of principal components for averaged algorithm PCA

Finally, we conducted principal component analyses to identify the most meaningful variables in the algorithm. When considering only the left ears, we initially accepted the first three principal components based on Kaiser’s rule and Cattell’s scree plot method (Fig. 5a). Principal component 1 explained 56.4% of the variance, principal component 2 explained 20.7% of the variance, and principal component 3 explained 10.9% of the variance for a total of 88.02% variance explained. However, in light of the findings regarding measurement error in the inner triangle most notably, we decided to retain only principal component 1, as it explained over half of the variance and was highly loaded by the curvature measures. The variables with the highest loadings in principal component 1 were included: C_R_3, C_R_4, and O3. The inner triangles measures were highly loaded in principal components 2 and 3. The factor pattern and loading values for the retained principal component of the left ear can be seen in Table 5.

**Table 5:** Principal component loadings for the three PCA analyses using orthogonal rotation

Left ear		Right ear			Both ears	
Variable	PC 1	Variable	PC 1	PC 2	Variable	PC 1
C_R_3	0.98	C_R_3	0.94	−0.07	C_R_3	0.97
C_R_4	0.95	C_R_4	0.93	0.26	C_R_4	0.95
C_R_3	0.93	C_R_5	0.89	0.40	C_R_5	0.91
C_R_2	0.90	C_R_6	0.84	0.50	O3	0.89
C_R_5	0.90	O2	0.80	−0.43	O2	0.88
O2	0.89	O3	0.76	−0.20	C_R_2	0.84
C_R_6	0.78	C_R_7	0.75	0.59	C_R_6	0.84
C_R_1	0.66	C_R_2	0.66	−0.63	C_R_7	0.69
O1	−0.89	O1	−0.80	0.15	O1	−0.88
I2	−0.16	C_R_1	0.35	−0.82	I2	−0.08
C_R_7	0.53	I2	0.22	0.33	I3	−0.25
I1	0.13	I3	−0.24	0.52	C_R_1	0.55
I3	−0.24	I1	0.56	−0.03	I1	0.34

**Fig. 6:**
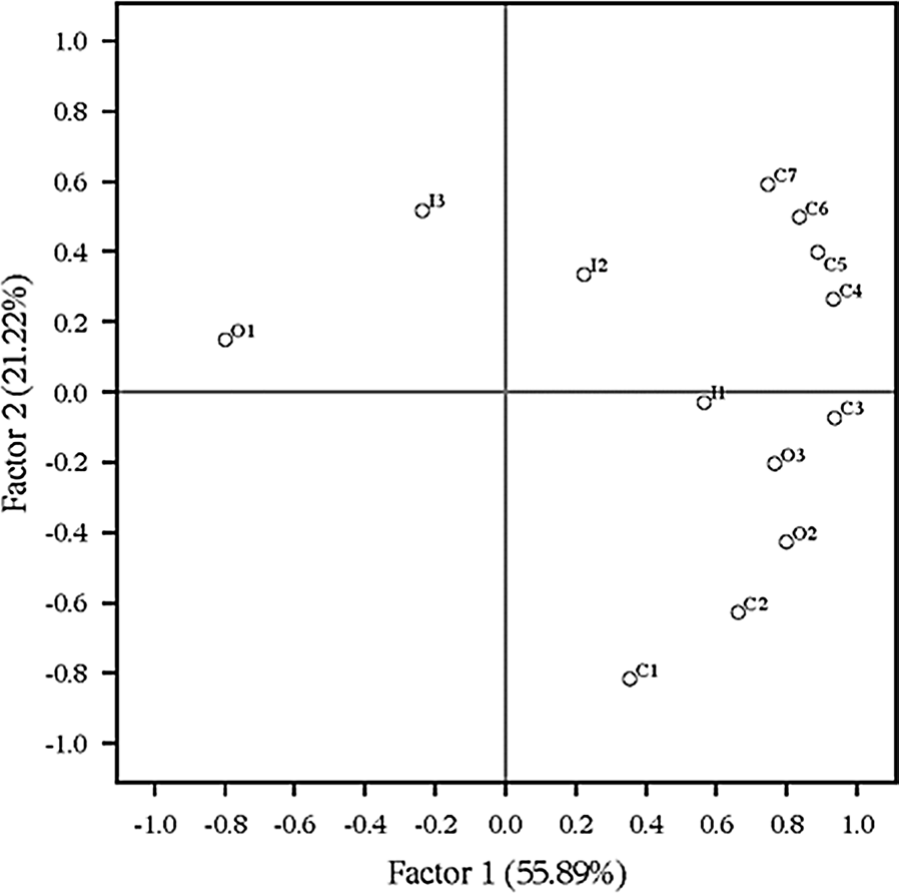
Bivariate *plot* depicting relationship between the retained components, PC1 and PC2, of the right ear algorithm

We conducted the same analysis for the right ear, initially accepting four principal components based on Kaiser’s rule and Cattell’s scree plot method (Fig. 5b). Principal component 1 of the right ears explained 51.1% of the variance, principal component 2 explained 19.4% of the variance, and principal component 3 explained 13.1% of the variance for a total of 83.6% variance explained. For the same reasons explained in the left ear PCA, only principal components 1 and 2 were retained, as principal components 3 and 4 were highly loaded by inner triangle variables. The variables with the highest loadings in principal component 1 were included: C_R_3, C_R_4, C_R_5, C_R_6, and O2. The curvature measures not accounted for in principal component 1 were loaded highest in principal component 2, including: C_R_1, C_R_2, C_R_7, C_R_6, and O2. The factor pattern and loading values for the retained components of the right ear can be seen in Table 5. The bivariate plot illustrating the correlation between principal components 1 and 2 can be seen in Fig. 6.

For our final principal component analysis, we first averaged the left and right algorithm variables into a thirteen variable algorithm representing both ears for each of the twenty-five individuals. This was to mirror the logic behind the re-identification analyses, where the underlying assumption was that in almost all scenarios, two ears would be available for the identification of each individual. The same criteria (Cattell and Kaiser) as for the previous analyses for selecting the principal components to retain was repeated (Fig. 5c), but for the same reasons in the left ear, only principal component 1 was retained, as principal component 2 was highly loaded by inner triangle variables. Principal component 1 accounted for 56.7% of the variability, and was most heavily loaded by C_R_3, C_R_4 and C_R_5. These findings were consistent with the independent analyses of the left and right ears. The loading values for the retained principal component of both ears can be seen in Table 5.

## Discussion

The health care systems of low- and middle-income countries are hampered by their inability to follow individuals consistently and accurately over time and space. Consequently, each health care encounter acts in isolation and longitudinally-focused global public health efforts, whether preventive or therapeutic, fail to have the desired and desperately needed impact on the target population. Through our analyses, we demonstrated that a simplified process of extracting biometrics data from a three-dimensional object has the potential for successful application in these health care settings. Successful health care begins with identification, and therefore, this study provides a platform for this important first step.

Our simplified analog process yielded high enough accuracy and replicability to result in correct re-identification amidst a pool of potential matches. We established the importance of sufficient measurement accuracy and reliance on anatomical ear structures that were consistently present yet sufficiently heterogeneous across individuals. We concluded that not all variables of our algorithm contributed to identification, some even seeming to hinder the process. Conversely, the contribution of data from both left and right ears abetted the process of identification. These findings support the potential of scaling biometrics systems for global health application.

Based on the analyses conducted, it was clear that different sections of our algorithm varied in measurement accuracy and in contribution to identification. Through our analysis of inter-investigator measurement precision, the inner triangle was found to have the greatest amount of measurement error associated with it. This led to high but uninformative standard deviation for the inner triangle variables across subjects, and was likely a result of high heterogeneity of the involved anatomical structures across individuals, e.g. the antitragus. Conversely, the curvature section as a whole was found to be the most accurately measured, but to have the smallest variation across subjects. However, the principal component analysis identified the curvature variables as consistently and unassailably accounting for the most variation across subjects. The final PCA, which evaluated the average between the left and right ear algorithms, identified C_R_3, C_R_4 and C_R_5 as the highest loaded variables (Table 5). In addition, the final PCA identified O3 and O2 as the fourth and fifth highest loaded variables. These two variables were also the most precisely measured across investigators, likely because the structures underlying these measures were consistently identified across the subjects.

Following the re-identification experiment, we conceptualized that the curvature variables were contributing the most to our own identification decision-making due to the likeness between investigators’ measurements. The principal component analysis was highly compatible with our empirical hypothesis. In conclusion, the curvature variables of this experimental algorithm had the least amount of measurement error, the smallest variation across subjects, but accounted for the greatest variability between subjects. In conclusion, we demonstrated both empirically and statistically that these variables contributed the most to identification. These analyses provide an excellent example of data noise versus signal. The algorithm was designed to be multifaceted with the purpose of accounting for variability in ear structure across individuals and to provide ample data for our analysis. We conclude that the efficiency and accuracy of identification would have been improved by the minimization of data points to the measurements most confidently made by investigators (i.e. reducing the noise). However, as demonstrated by our re-identification analyses, the ability to leverage both left and right ears (and as a result, increasing the data points) during the process of identification in fact increased accuracy to 100%. The re-identification experiments provided confirmation of our statistical findings, but also important context for the development of an ear biometrics identification application.

It is worth going back to the discussion of ‘sufficient’ accuracy. In the majority of biometrics applications, researchers must apply the concept of 1: N verification, that is that identification is made on the basis of comparing one person to N others to ascertain identity. If designed and implemented appropriately, the automated biometrics system built into the Smartphone application would eliminate this issue through multiple measures. First, while most biometrics systems attempt to identify at the population level, a more appropriate approach for our setting of interest would be to either focus on smaller units of the population (e.g., the patients under the purview of one community health worker or one rural health clinic’s catchment area) or to create a system that could systematically interrogate hierarchical tiers of a patient database in the event of no match. Under the second approach, the system would first interrogate the most local pool of identities (e.g. the local clinic), and only if unable to make a match would the system query the next tiers (e.g. an adjacent clinic, the district, the region, etc.). As a result the pool of potential matches would be minimized in most scenarios. Second, the application would provide a number of rank-ordered matches, therefore allowing the health care worker to select the correct identity based on information obtained from the patient. This expands the accuracy of the application, by allowing external information to support selection of the correct match. Additionally, the input of other identifiers into the query would dramatically reduce the pool of possibilities. Sex, for instance, would effectively reduce the pool in half. Lastly, the system would learn longitudinally, updating the stored biometrics data with each health care visit. This would be important for making the system robust against the slowly but steadily changing dimensions of a growing ear. Although under certain circumstances (i.e. a new patient), the system would be required to perform 1: N identification, under most circumstances it would only have to perform segmented identification, that is, obtaining identity through biometrics and then confirming with a verbal query regarding patient information.

As a proof of concept study, the results should be interpreted in light of other complex biometrics research investigating the feasibility of ears for identification. Once developed into a pilot program, the application would likely take advantage of more complex image processing and pattern recognition algorithms to increase accuracy. Additionally, this cross-sectional study looked at adult ears. The true potential benefit of ear biometrics identification is with children. Worldwide, the births of approximately 230 million children <5 years have gone unregistered, including more than 80% of births in sub-Saharan Africa [36]. These children go untracked by national health systems. As the primary causes of under-five mortality are preventable, the global health community has an invested interest in improving continuity of care [37]. There is therefore a need for a longitudinal study with infants and/or young children to investigate if ear biometrics are robust against periods of rapid growth and development, namely in the first few years of life. An additional potential limitation of ear biometrics in the context of global health is cultural modification to the ear, including piercings and cuttings, often related to coming of age in various cultures. To evaluate these concerns, we have initiated a longitudinal birth cohort study to capture ear development over the first six months of life.

Although two dimensional technology was assessed in this proof of concept study and currently appears the most accessible for mHealth applications, there are promising technologies on the horizon that may allow for such an application to take advantage of the higher accuracy and more robust nature of three dimensional imaging. Such an advance may eliminate many of our current challenges, including ensuring a standardized image capture process, illumination, and potentially accuracy.

## Conclusion

Although a simplified ear biometrics system for patient identification may be unfeasible at the population level, the potential for such an application to aid health care systems within smaller geographical settings, local clinic populations, and longitudinal studies merits further investigation (Additional file 1: Table S1). A Smartphone application able to capture succinct and simple biometrics data from ears to aid in patient identification in remote global settings has the capacity to lend great advances to improving health outcomes. As the true benefit of such an application would be realized in the realm of neonatal and child health in global health contexts, future research should aim to deepen our understanding of ear growth during the first years of life within the context of biometrics application. Our next steps are to use this foundation to inform the development of our pilot Smartphone application. This application, in conjunction with a Smartphone cradle to help standardize the image capturing process and ambient lighting, will be piloted on a longitudinal cohort of young infants in the coming year. We are entering an era where Smartphone’s and similar technology are becoming commonplace around the world, and there is hope that increased interest in capitalizing on this phenomenon will aid in bringing the improvement of global health outcomes to match a similar trend.

## Additional file

**Additional file 1: Table S1.** Ear biometrics dataset.
